# Delirium Tremens

**Published:** 1882-07

**Authors:** 


### DELIRIUM TREMENS.

At a recent meeting of the Cambridge (Eng.) Medical Society (*Lancet*), Dr. Latham introduced the subject of the treatment of delirium tremens. He thought the subject offered scope for considerable difference of opinion, and was a suitable one for general discussion. He referred, first, to sleeplessness as a prominent symptom, and said that if sleep were obtained, recovery generally ensued quickly. Was the induction of sleep, then, a *sine qua non* in treatment? If sleep did not ensue, subarachnoid effusion and coma were likely to follow in some cases, while the majority might recover; but Dr. Latham thought the disease ought to be treated actively, and that dangerous remedies might be used with advantage to patients. He divided cases of delirium tremens into three classes: (1) Patients in moderate health who had used stimulants in excess; (2) those in robust health who had indulged after excitement or distress, chiefly young men; and (3) those broken down in health, and with damaged organs. The first class he thought could be treated with opium, without risk, provided the urine was free from albumen; and he had been surprised to find albumen in the urine in a large proportion of cases without any other indications of kidney disease, and the albumen disappearing as the patient recovered. If albuminuria existed, the use of opium or morphia could only be pernicious. The first thing to do was to give soup, beef-tea, and alcohol in nearly the accustomed doses for twenty-four hours, and then opium; having previously given, if necessary, a dose of calomel. He advised the hypodermic injection of morphia, first in half-grain doses, and then in doses of one quarter grain every half hour till sleep was procured or the patient was distinctly under its influence, as shown by the condition of the pupil. He believed diminution in quantity of stimulants taken by the patient to be an early symptom, and not a cause of the disease. In the second class he advised, not opium, but henbane. He had given bromide of potassium in forty-grain doses every four hours, and found the tincture of henbane in doses of one drachm every four hours succeeded better. He also thought that Merck's preparation of hyoscyamine would be useful, but had not yet given it a trial. In the third class he thought opium should be given with great caution, and that the free administration of stimulants was important, even as large doses as those customary with the patient. He mentioned a case in which a gentleman, in his eager desire for insensibility, had taken 240 grains of chloral and the same quantity of bromide of potassium at a dose, and survived after very free action of the skin, bowels, and kidneys. He deprecated the use of large doses of digitalis, as suggested by Dr. Jones, of Jersey. Mr. T. Hyde Hills, speaking from his experience as a jail surgeon, thought leaving off stimulants was a cause of the disease, and that many prisoners became delirious after admission, owing to this deprivation. He advised giving the usual amount of alcohol to which the prisoner had been accustomed, and thought chloral of benefit. Mr. Hodson thought that the combination of chloral and capsicum was useful. Dr. Smith alluded to the usefulness of cold sponging of the body as a means of inducing sleep. Dr. Latham, in reply, stated that he recognized the capsicum in confirmed cases ; it was much esteemed in India. In young men he approved of Dr. Graves' plan of giving antimony and opium every two hours.—*Med. and Surg. Rep.*

